# Leukocyte-Rich Platelet-Rich Plasma Improves Cartilage Repair After High Tibial Osteotomy: A Second-Look Arthroscopic Study

**DOI:** 10.3390/biomedicines14081664

**Published:** 2026-07-24

**Authors:** Jesse Chieh-Szu Yang, Yu-Hung Tian, En-Rung Chiang, Yu-Ping Su

**Affiliations:** 1 Department of Surgery, School of Medicine, National Yang Ming Chiao Tung University, Taipei 112304, Taiwan; 2 Department of Orthopaedics and Traumatology, Taipei Veterans General Hospital, Taipei 112201, Taiwan

**Keywords:** high tibial osteotomy, platelet-rich plasma, leukocytes, cartilage regeneration, osteoarthritis, second-look arthroscopy

## Abstract

**Background**: High tibial osteotomy (HTO) is commonly performed to manage medial compartment knee osteoarthritis by correcting mechanical alignment; however, the role of adjunctive regenerative therapies remains uncertain. **Methods**: This retrospective study compared leukocyte-rich platelet-rich plasma (LR-PRP) with leukocyte-poor PRP (LP-PRP) in patients undergoing HTO. Forty patients were allocated into three groups: HTO alone (*n* = 10), HTO with LR-PRP (*n* = 20), and HTO with LP-PRP (*n* = 10). Clinical outcomes were assessed preoperatively and at 12 months using the Visual Analog Scale, Oxford Knee Score, and Western Ontario and McMaster Universities Osteoarthritis Index. Cartilage repair appearance was evaluated through second-look arthroscopy using the ICRS grading and Koshino staging systems. Multivariable analysis of covariance (ANCOVA), adjusting for baseline imbalances, was employed to evaluate postoperative outcomes. **Results**: All groups demonstrated significant improvements in pain and function (*p* < 0.05), with no significant differences among groups. However, Group B exhibited a greater shift toward lower ICRS grades than Group A (*p* < 0.05), whereas no significant difference was found between Groups C and A. Arthroscopic findings revealed more complete defect coverage and improved structural integrity in the LR-PRP group. **Conclusions**: These findings demonstrate a clear discrepancy exists between clinical and structural outcomes; while HTO drives substantial and comparable short-term functional improvements across all cohorts, adjunctive LR-PRP is positively associated with a significantly enhanced arthroscopic cartilage repair appearance compared to LP-PRP or HTO alone. Further prospective studies are needed to validate these findings and elucidate the underlying biological mechanisms.

## 1. Introduction

Knee osteoarthritis (OA) is a complex cellular and tissue pathology involving the entire joint structure; it is primarily characterized by articular cartilage degradation but also encompasses subchondral bone remodeling, osteosclerosis, osteophyte formation, synovial hyperplasia, and pathological alterations in other periarticular tissues [1]. Driven by multifactorial etiologies—including cumulative mechanical wear, aging, trauma, and joint malalignment—OA is a chronic, disabling disease clinically defined by three hallmark features: pain, transient stiffness, and progressive functional limitation [2]. Although traditionally associated with older adults, chronic knee inflammation is increasingly observed in younger populations due to prolonged poor posture or high levels of physical activity [3,4]. In advanced stages, total knee arthroplasty (TKA) may be necessary. However, for patients with varus deformity-induced OA [5] or those seeking to preserve the native joint, high tibial osteotomy (HTO) represents a viable alternative. HTO enables joint preservation while avoiding the inherent risks and complications associated with prosthetic replacement [6].

HTO is primarily indicated for medial compartment osteoarthritis and varus deformity. The procedure employs specialized osteotomy saws and blades to accurately control the location and angle of the bone cut. By realigning the mechanical axis of the tibia, it effectively redistributes load away from the medial compartment, thereby reducing stress on the articular cartilage and limiting further degeneration [7].

The survival rate is a key metric for assessing the long-term efficacy of HTO, providing clinicians and patients with essential prognostic information for postoperative management. Current evidence suggests that modern HTO techniques achieve a 10-year survival rate exceeding 50% [8,9]. Accordingly, identifying strategies to improve postoperative durability, enhance cartilage repair, and relieve arthritic symptoms remains an active area of research [10].

Recent advances in regenerative medicine, including platelet-rich plasma (PRP) and mesenchymal stem cells (MSCs), have been investigated as adjunctive therapies to improve HTO outcomes and survival rates [11]. PRP is characterized by a high concentration of platelets containing various growth factors [12], including platelet-derived growth factor (PDGF), vascular endothelial growth factor (VEGF), transforming growth factor-beta (TGF-β), and epidermal growth factor (EGF). PRP has been widely utilized as an adjunctive therapy in knee osteoarthritis surgery to alleviate patient pain and enhance motor function [13,14], as well as in high tibial osteotomy (HTO) to promote structural cartilage repair [15,16]. However, variability in clinical outcomes persists due to differences in PRP preparation protocols and collection methods, leading to variations in leukocyte content. Some studies suggest that leukocyte-poor PRP (LP-PRP) may limit exposure to inflammatory mediators; conversely, leukocyte-rich PRP (LR-PRP) has been shown to facilitate angiogenesis and extracellular matrix deposition, particularly in the context of tendon and bone healing [17]. Nonetheless, the precise biological role of leukocytes within PRP remains highly controversial and largely unresolved [18,19]. Therefore, this retrospective study focuses on knee cartilage regeneration by comparing the effects of leukocyte-rich PRP (LR-PRP) and leukocyte-poor PRP (LP-PRP) as adjunctive therapies following HTO.

## 2. Materials and Methods

### 2.1. Patient Selection and Study Design

**Figure 1:**
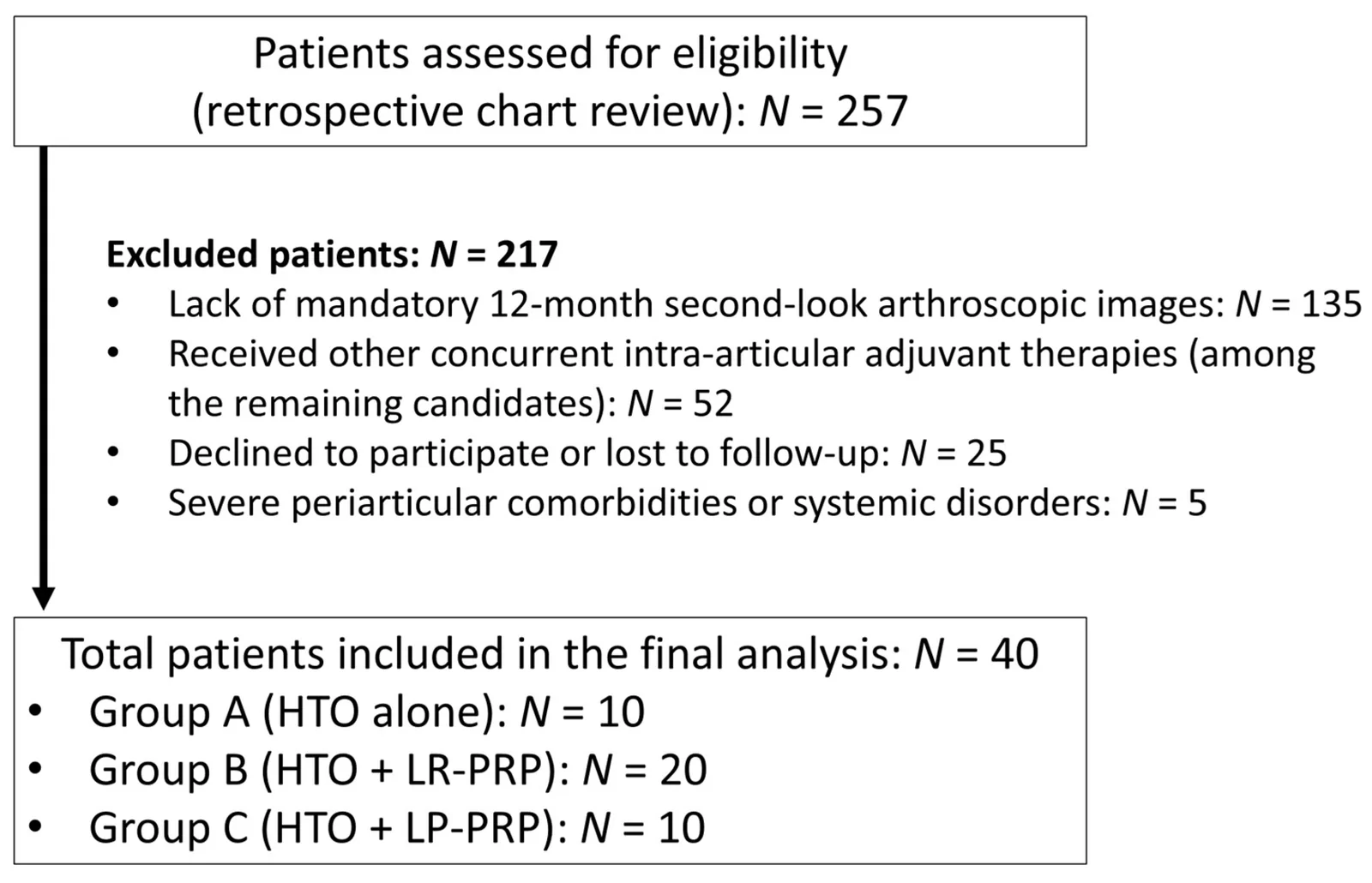
Flowchart of Patient Screening, Exclusion, and Allocation. Note that sub-category reasons for exclusion are not mutually exclusive due to overlapping clinical criteria in the retrospective database. A final cohort of 40 patients met all eligibility criteria and was allocated into three groups.

This retrospective study was approved by the Institutional Review Board of Taipei Veterans General Hospital (IRB No. 2024-02-008ACF) on 29 February 2024, and informed consent was obtained from all participants. We reviewed records of patients aged ≥20 years who underwent HTO at Taipei Veterans General Hospital between 1 May 2016, and 15 December 2023, with a minimum follow-up of 12 months. To be included in this study, all patients must have undergone both the initial surgery and a subsequent second-look arthroscopy during the follow-up period. Patients were excluded if they declined participation, had systemic conditions affecting mobility or physical function, had cognitive impairment limiting communication, or received additional adjuvant therapies after HTO. The detailed patient screening, inclusion/exclusion process, and subsequent grouping are illustrated in the flowchart (Figure 1). This study was reported following the Strengthening the Reporting of Observational Studies in Epidemiology (STROBE) statement (Table S1).

The cohort was stratified into three groups based on postoperative management: Group A (control) underwent HTO alone; Group B (LR-PRP) received HTO with LR-PRP; and Group C (LP-PRP) received HTO with LP-PRP. Baseline characteristics, including age, sex, weight, height, and preoperative International Cartilage Regeneration & Joint Preservation Society (ICRS) and Kellgren–Lawrence (K-L) grades, were documented to ensure comparability among groups.

### 2.2. Surgical Procedure

All procedures were performed by a single senior surgeon to ensure consistency. Before HTO, diagnostic arthroscopy was performed to assess intra-articular cartilage damage. The medial opening-wedge HTO was executed using a biplanar osteotomy technique, specifically preserving the tibial tubercle to maintain structural stability. The osteotomy site was expanded at the medial cortex according to pre-planned corrections, ranging from 10.0 to 15.9 mm in wedge height based on individual patient anatomy. Notably, no autograft, allograft, or bone substitutes were filled into the osteotomy defect, relying entirely on the rigid fixation of the implant. To maintain the alignment correction and ensure rigid initial fixation, a contoured titanium alloy Medial High Tibial Plate (MHTP; TomoFix, DePuy Synthes, Paoli, PA, USA) was locked into place using eight 5.0 mm locking screws.

**Figure 2:**
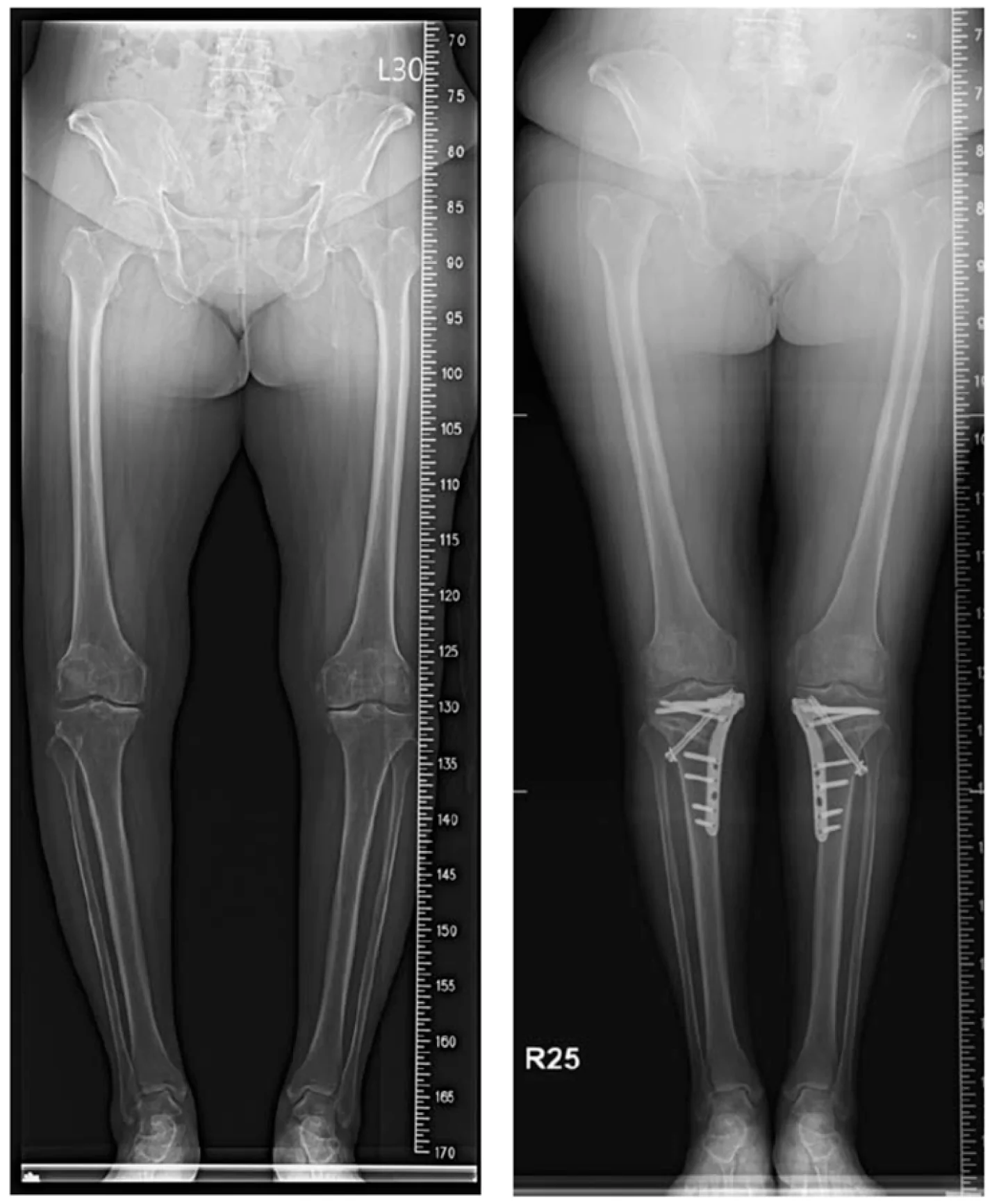
X-ray of opening-wedge valgus HTO. The left image is an X-ray taken before the surgery, and the right image is an X-ray taken after the surgery.

Preoperative and postoperative radiographic parameters were meticulously tracked using digital long-limb standing radiographs. (Figure 2) The target alignment was set to achieve a mechanical axis shifting to a target weight-bearing line (WBL) of 62.5% (the Fujisawa point) through the lateral knee compartment. Accordingly, the mean preoperative hip–knee–ankle (HKA) angle was corrected to a mean postoperative HKA angle of 2.4° to 2.5° of valgus, achieved via an operative correction angle ranging between 13.2° and 15.9°. The medial proximal tibial angle (MPTA) subsequently adjusted from a varus baseline to a mean postoperative range of 96.3° to 98.2°. The posterior tibial slope was carefully controlled and maintained between 5° and 7° during the biplanar cut to avoid any sagittal malalignment. No additional intra-articular procedures (such as meniscectomy or microfracture) were performed concurrently to isolate the therapeutic effect of HTO and PRP.

Postoperatively, no casts or immobilization devices were required. A standardized rehabilitation protocol was implemented for all cohorts: patients were encouraged to perform passive range-of-motion exercises and initiate partial weight-bearing as tolerated on the first postoperative day using crutches. Progression to full weight-bearing was typically achieved by 6 weeks, depending on radiographic signs of bone healing at the osteotomy gap. Return to sports was generally permitted between 3 and 6 months. A second-look arthroscopy was conducted 12 months after HTO during scheduled hardware removal to evaluate cartilage repair appearance.

### 2.3. PRP Preparation and Injection Protocols

In Group B, LR-PRP was prepared using an automated blood component separation system (Phoenix, Safetran BioMedical Inc., Taipei, Taiwan). Sixty milliliters of whole blood were centrifuged with optical detection for 6 min, yielding 6 mL of LR-PRP. This preparation achieved an absolute platelet concentration ranging from 8.0 × 10^5^ to 1.2 × 10^6^ cells/μL (approximately 2.5–4× baseline), an absolute leukocyte concentration of 1.5 × 10^4^ to 2.2 × 10^4^ cells/μL (with monocytes enriched to 3× baseline alongside granulocytes), and residual red blood cells controlled at approximately 7% of physiological whole blood levels.

In Group C, LP-PRP was prepared using the Arthrex ACP Double-Syringe System (Arthrex, Inc., Naples, FL, USA) and a centrifuge (Rotofix 32 A, Andreas Hettich GmbH & Co. K., Tuttlingen, Germany). Fifteen milliliters of whole blood were processed for 5 min to obtain 4–7 mL of LP-PRP. This leukocyte-poor formulation yielded an absolute platelet concentration of 3.5 × 10^5^ to 5.5 × 10^5^ cells/μL (1.2–2.2× baseline), while the absolute leukocyte count was drastically depleted to approximately 1.2 × 10^3^ cells/μL (0.3× baseline) and red blood cells were minimized to 0.004× physiological levels [12].

Following separation, both types of PRP were administered immediately without any chemical activation (e.g., calcium chloride or thrombin) to avoid altering natural growth factor release kinetics. In both groups, PRP was administered intra-articularly on the first postoperative day. Due to economic and financial considerations under local healthcare coverage, the vast majority of our patients chose to receive a single PRP injection; hence, this study focused exclusively on patients adhering to this single-dose regimen to ensure cohort homogeneity. Notably, no adverse events, severe pain flares, or complications related to PRP administration were reported in any patient during the entire 12-month follow-up period.

### 2.4. Clinical and Cartilage Evaluation

Clinical outcomes were assessed preoperatively and at 12 months after HTO. Pain was measured using the Visual Analog Scale (VAS) [20], whereas the Oxford Knee Score (OKS) [21] was used to evaluate symptoms (7 items) and physical function (5 items). The Western Ontario and McMaster Universities Osteoarthritis Index (WOMAC) [22] provided a subjective assessment of pain, stiffness, and functional limitations. Cartilage status was graded using the ICRS scale [23,24], ranging from Grade 0 (normal) to Grade 4 (complete loss). Cartilage regeneration was further assessed using the Koshino staging system [25], which classifies findings from no regenerative changes (Grade 1) to complete fibrocartilage coverage (Grade 4).

To ensure objectivity and minimize bias, all arthroscopic images recordings were independently evaluated by two experienced orthopedic surgeons. To enhance the blinding protocol, one senior surgeon was completely blinded to the patient treatment allocation (Group A, B, or C) during the entire grading process. The inter-observer agreement between the two evaluators was highly consistent across most cases; in any rare event of a grading discrepancy, a final consensus score was reached through a collaborative review and mutual discussion between both surgeons. No formal mathematical inter-observer reliability analysis (such as a Kappa statistic) was additionally performed due to the retrospective nature of this study.

### 2.5. Statistical Analysis

Statistical analyses were performed using SPSS version 24 (SPSS Inc., Chicago, IL, USA). A *p*-value < 0.05 was considered statistically significant. Continuous variables (VAS, WOMAC, and OKS) are expressed as means ± standard deviations and exact *p*-values, 95% confidence intervals (95% CIs), and effect sizes are reported to determine clinical relevance. Initial group comparisons were performed using one-way analysis of variance (ANOVA) followed by post hoc pairwise *t*-tests with Bonferroni correction. To rigorously account for baseline discrepancies among cohorts—including significant differences in preoperative cartilage lesion severity, patient age, sex, and body mass index (BMI)—multivariable ANCOVA models were additionally constructed to calculate baseline-adjusted estimates.

Ordinal variables (ICRS and Koshino scores) are presented as medians and interquartile ranges (IQRs) and were analyzed using the Kruskal–Wallis test. When significant differences were detected, post hoc pairwise comparisons were conducted using the Mann–Whitney U test with Bonferroni correction to control for multiple comparisons. Categorical variables were analyzed using the chi-square test or Fisher’s exact test, as appropriate. Given the retrospective nature and relatively limited sample size of this study, an a priori sample size calculation was not conducted. Consequently, this study was treated as an exploratory analysis, and a cautious interpretation was applied regarding statistical power in negative findings.

## 3. Results

### 3.1. Patient Demographics and Baseline Characteristics

A total of 257 patient charts were initially screened for eligibility. Based on our predefined strict criteria, 217 patients were excluded due to overlapping confounding factors—predominantly the lack of 12-month second-look arthroscopy or the receipt of other concurrent adjuvant therapies—leaving a final study cohort of 40 patients. The comprehensive, step-by-step screening and allocation pathway is rigorously detailed in the study flowchart (Figure 1). During enrollment, Group B included 20 patients. In contrast, Group A comprised 10 patients and Group C also included 10 patients. This uneven group distribution was a direct consequence of strict retrospective screening, where several otherwise eligible candidates were excluded due to the receipt of other adjuvant therapies or the lack of mandatory 12-month second-look arthroscopic images required for objective cartilage evaluation. We explicitly acknowledge this relatively small and unbalanced sample size as a structural limitation of our study design.

The baseline demographics and clinical characteristics of the three groups are summarized in Table 1. At baseline, no statistically significant differences were observed among the three groups in terms of age, height, weight, BMI, sex, or K-L grade (*p* > 0.05). Furthermore, all patients demonstrated intact anterior cruciate ligament (ACL) and posterior cruciate ligament (PCL) function, with no cases of ligamentous injury or rupture documented. However, significant baseline discrepancies were identified in preoperative objective and subjective parameters; specifically, the Group C demonstrated significantly lower baseline ICRS scores (indicating less baseline cartilage damage) and worse preoperative OKS compared to the other groups (*p* < 0.05). To thoroughly neutralize the confounding effects of these baseline variations, multivariable ANCOVA models adjusting for age, sex, BMI, and initial status were rigorously employed for all subsequent post-operative functional analyses.

### 3.2. Pain and Functional Outcomes

**Figure 3:**
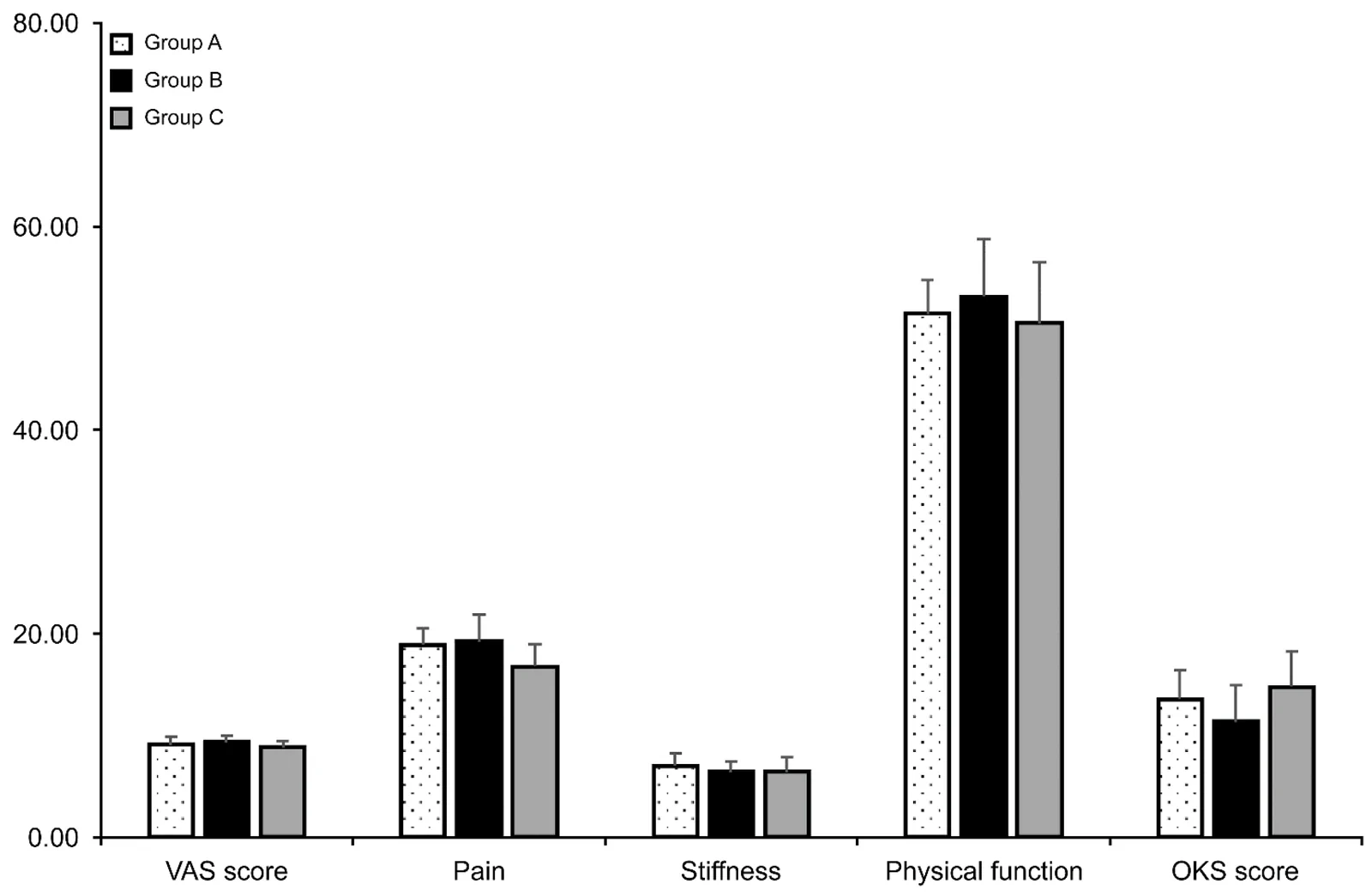
Evaluation of knee function before high tibial osteotomy.

**Figure 4:**
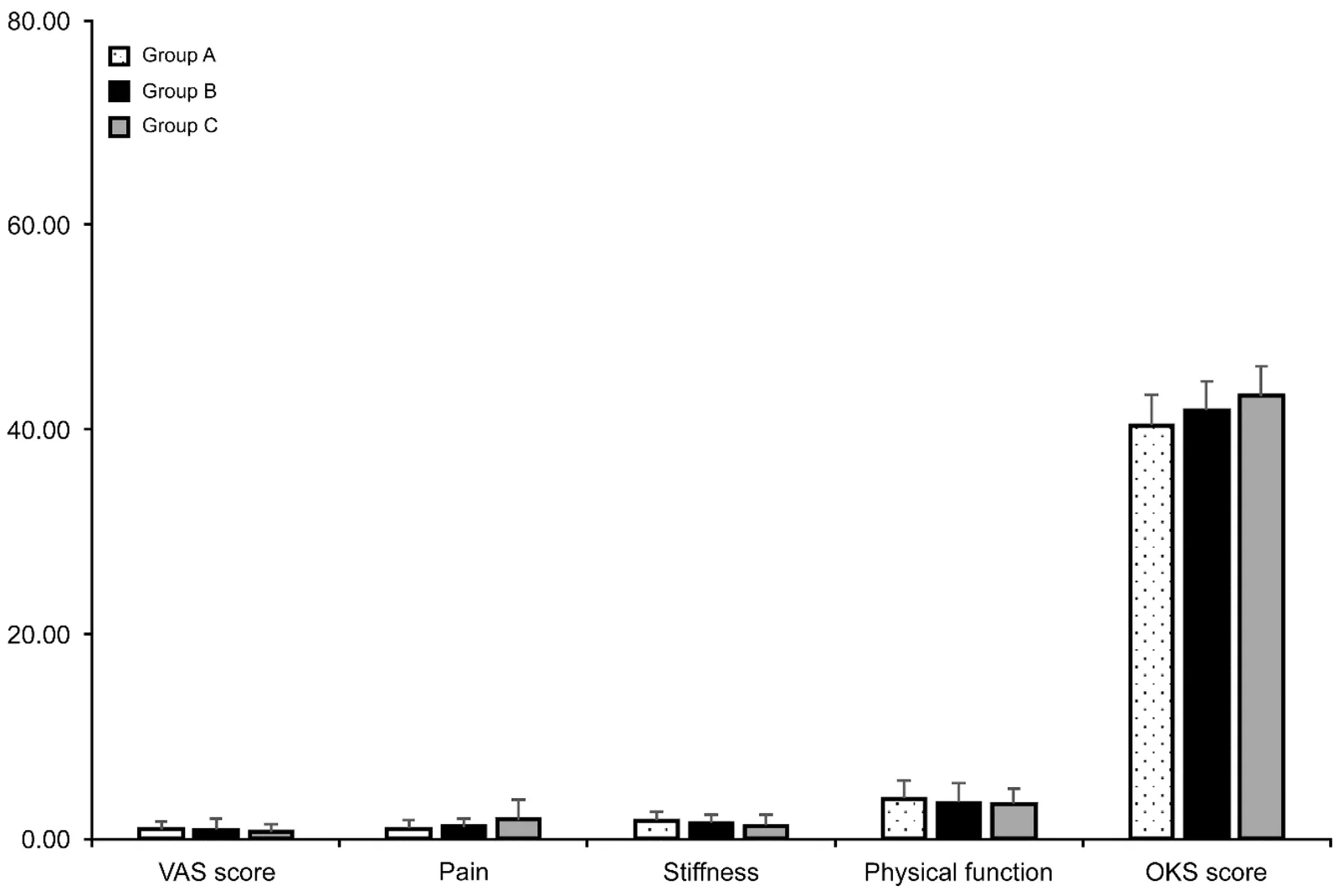
Evaluation of knee function 12 months after high tibial osteotomy.

At the 12-month postoperative milestone, all three treatment cohorts demonstrated substantial, clinically meaningful improvements in subjective pain relief and joint functional scores compared to their respective preoperative baselines (p<0.05) (Table 2, Figure 3 and Figure 4). Specifically, VAS and WOMAC pain scores in Group A improved from 9.1 ± 0.74 and 18.9 ± 1.66 to 0.9 ± 0.74 and 1.0 ± 0.82, respectively. Similarly, Group B exhibited improvements from 9.35 ± 0.67 and 19.25 ± 2.67 to 0.85 ± 1.14 and 1.15 ± 0.75. Group C scores improved from 8.8 ± 0.63 and 16.8 ± 2.2 to 0.6 ± 0.7 and 1.7 ± 1.77. To rigorously evaluate the therapeutic contribution of adjunctive PRP while controlling for baseline variances (e.g., the worse preoperative OKS in Group C), multivariable ANCOVA models were executed. After adjusting for age, sex, BMI, and preoperative baseline parameters, the adjusted statistical analysis demonstrated that there were no statistically significant differences among the three groups regarding 12-month postoperative VAS scores (adjusted *p* = 0.9541, partial *η*^2^ = 0.0029), WOMAC Pain sub-scores (adjusted *p* = 0.5198, partial *η*^2^ = 0.0401), or final OKS values (adjusted *p* = 0.198, partial *η*^2^ = 0.0963). These findings clearly demonstrate that while open-wedge HTO is a highly potent intervention for relieving mechanical overload and symptoms, the adjunctive intra-articular administration of either LR-PRP or LP-PRP did not yield an additional, statistically measurable subjective clinical benefit at the 12-month follow-up window, as evidenced by the very small effect sizes (partial *η*^2^ < 0.06 for most measures).

### 3.3. Arthroscopic Evaluation of Cartilage Repair Appearance

Cartilage damage was evaluated using the ICRS grading system. Preoperatively, most patients across all groups presented with Grade 3 or 4 lesions, indicating advanced chondral damage. Groups A and B had a higher proportion of Grade 4 defects, whereas Group C consisted predominantly of Grade 3 lesions (Table 3). No statistically significant differences in preoperative ICRS grade distribution were found between Groups A and B, whereas Group C exhibited relatively less severe cartilage damage at baseline.

At the 12-month second-look arthroscopy, all groups showed improvement in cartilage status, reflected by a shift toward lower ICRS grades. However, non-parametric multivariable analysis demonstrated that Group B achieved a remarkably superior structural cartilage repair appearance compared to both Group A and Group C (*p* < 0.0001). Specifically, 65% of patients in the Group B achieved an ICRS Grade 1 (near-normal tissue architecture), whereas 0% of patients in Group A and Group C achieved this degree of structural restoration. Conversely, 50% of Group A and 40% of Group C patients still exhibited persistent, uncovered severe ICRS Grade 3 defects at the 12-month mark.

Cartilage regeneration was further assessed using the Koshino staging system (Table 4). A greater proportion of patients in Group B achieved significantly higher tissue coverage and continuity compared to the other two groups. In contrast, Groups A and C predominantly remained at intermediate stages of regeneration.

**Figure 5:**
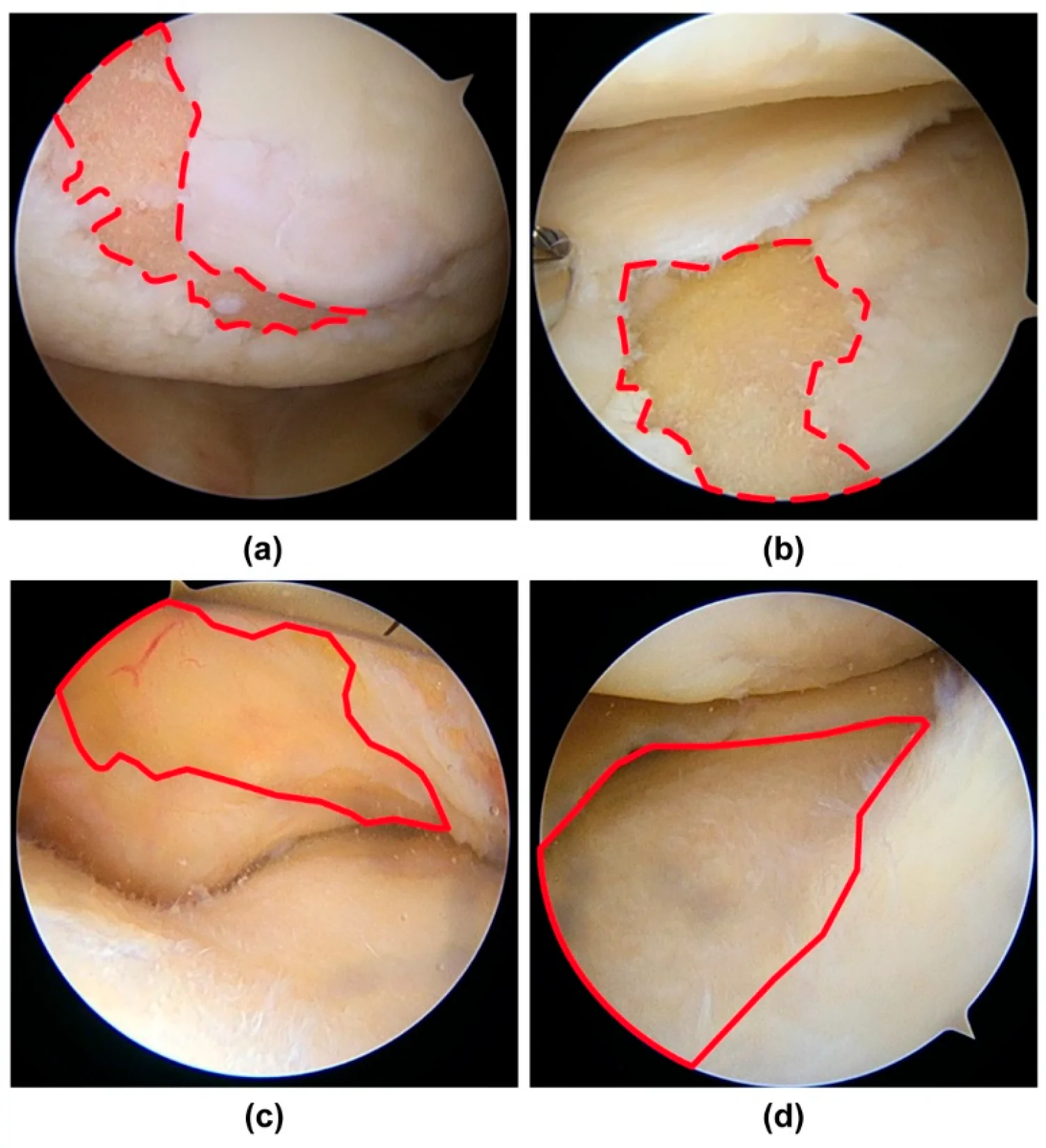
Arthroscopy images in Group A (only HTO). (**a**,**b**) are arthroscopic views at the initial surgery, with the dashed lines indicating the cartilage defect area. (**c**,**d**) are arthroscopic views at second-look arthroscopy, with the solid lines delineating the margins of repaired area.

**Figure 6:**
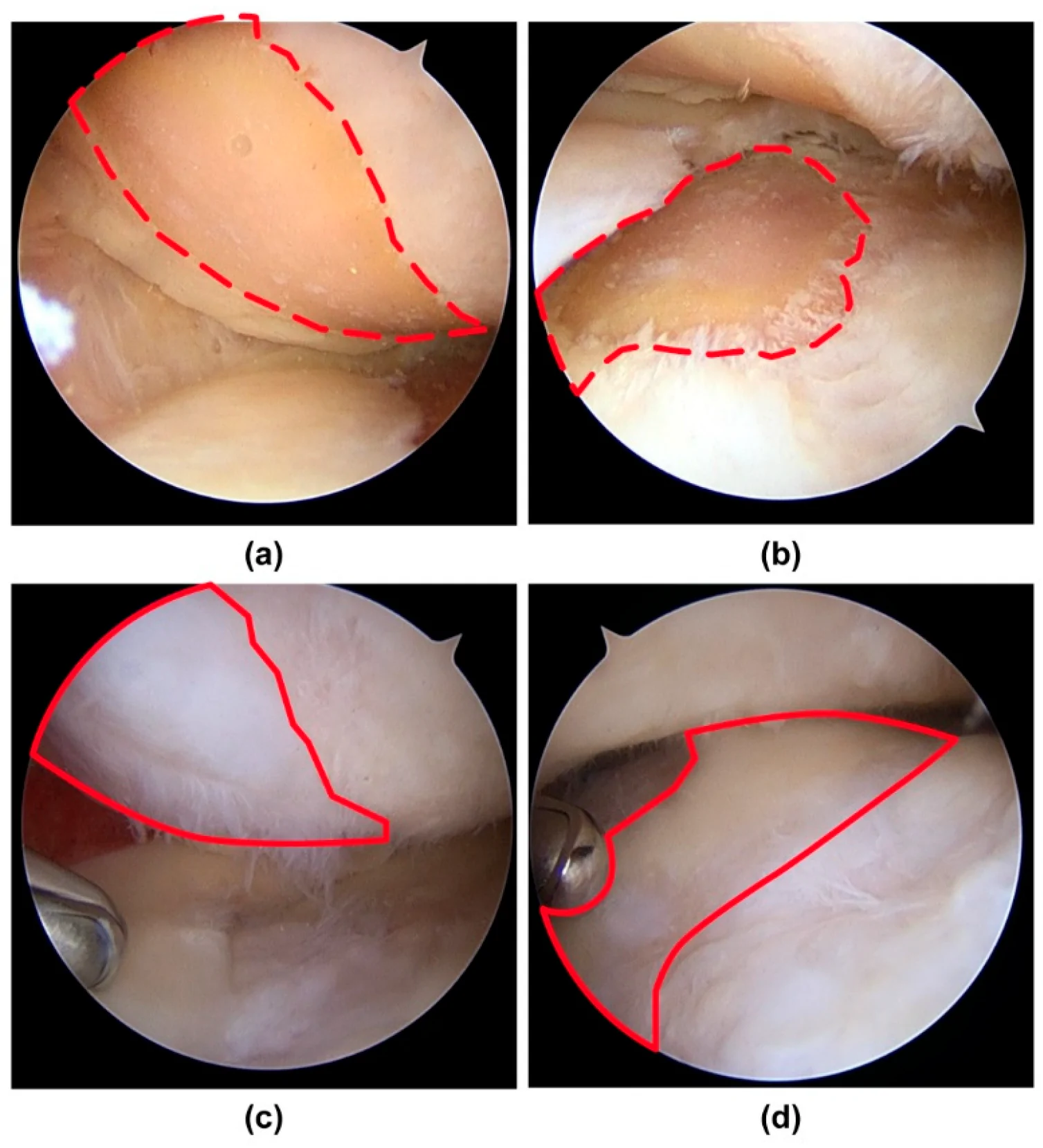
Arthroscopy images in Group B (HTO plus LR-PRP). (**a**,**b**) are arthroscopic views at the initial surgery, with the dashed lines indicating the cartilage defect area. (**c**,**d**) are arthroscopic views at second-look arthroscopy, with the solid lines delineating the margins of repaired area.

**Figure 7:**
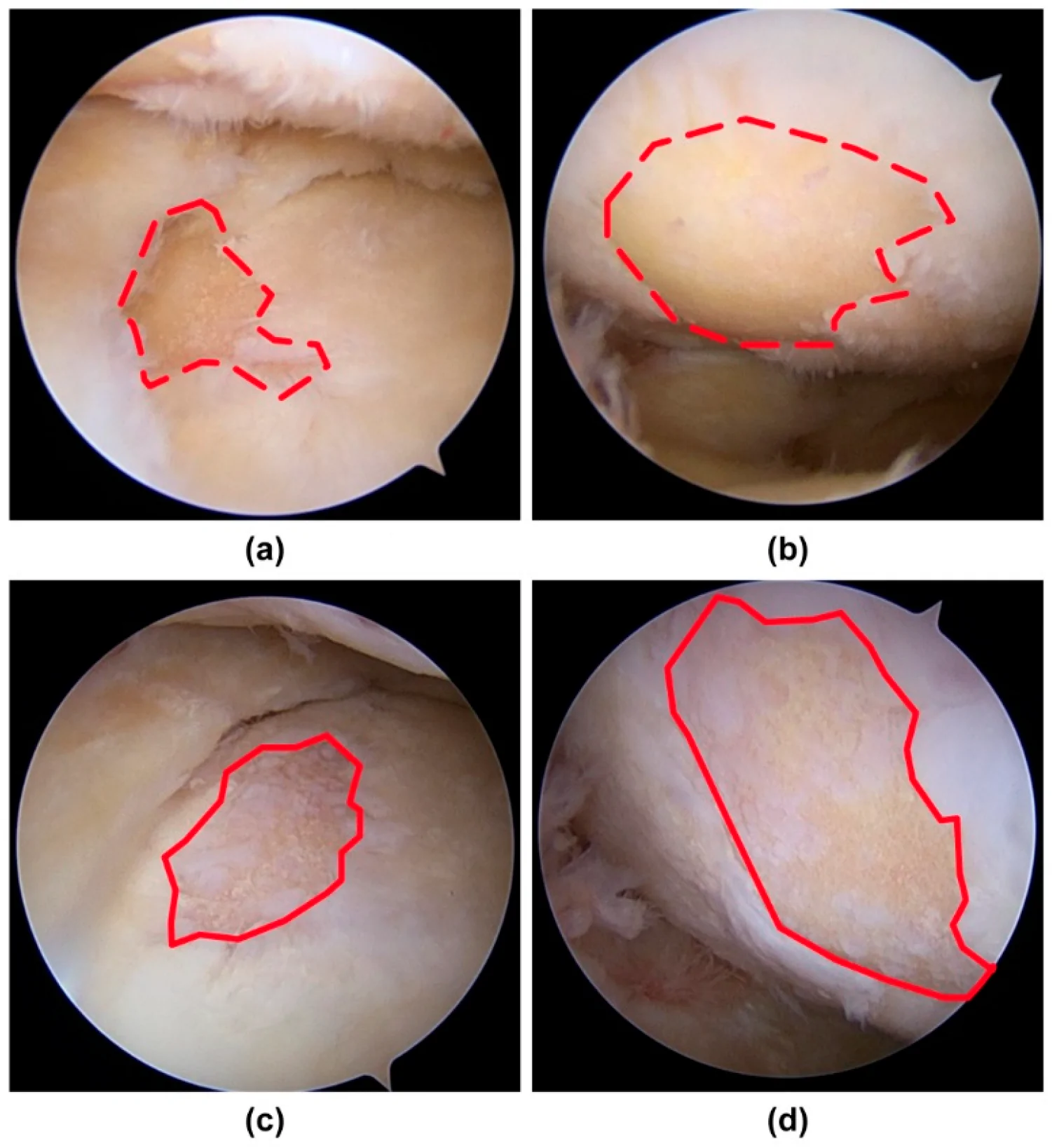
Arthroscopy images in Group C (HTO plus LP-PRP). (**a**,**b**) are arthroscopic views at initial surgery, with the dashed lines indicating the cartilage defect area. (**c**,**d**) are arthroscopic views at second-look arthroscopy, with the solid lines delineating the margins of repaired area.

Arthroscopic findings at 12 months were consistent with these grading results (Figure 5, Figure 6 and Figure 7). In Groups A (Figure 5) and C (Figure 7), the arthroscopic cartilage repair appearance was strictly limited, characterized by sparse, thin fibrocartilaginous tissue clusters or extensive bare bone exposure. In sharp contrast, Group B (Figure 6) demonstrated extensive and robust tissue remodeling, with thick, glossy white cartilaginous tissue covering the designated osteotomy defects more completely, presenting superior structural integration and tissue continuity. Taken together, these data provide strong evidence that the adjunctive application of LR-PRP is highly associated with an enhanced arthroscopic appearance of cartilage repair following HTO, substantially outperforming LP-PRP or HTO alone.

## 4. Discussion

The present study demonstrates that HTO significantly reduces knee pain, as evidenced by the marked decrease in postoperative pain and the robust improvement in functional scores across all groups (Table 2). Preoperatively, there was a baseline imbalance where Group C exhibited worse functional scores (OKS and WOMAC total) but milder baseline cartilage damage (ICRS grade) compared to Group B. However, after employing rigorous multivariable ANCOVA adjustments, we confirmed that this initial discrepancy exerted negligible influence on the 12-month postoperative subjective outcomes. These findings align with previous literature [3,26,27], confirming that HTO improves the biomechanical environment by realigning the tibial mechanical axis. This load redistribution decreases excessive stress on the medial compartment, thereby alleviating pain caused by repetitive joint friction.

Despite the uniform and excellent clinical improvements observed across all three groups, we identified a highly intriguing discrepancy between subjective clinical outcomes and objective structural repair. Clinically, postoperative VAS, WOMAC, and OKS scores showed no statistically significant differences among the groups, accompanied by very low effect sizes (Table 2). This lack of short-term subjective superiority suggests a “ceiling effect,” wherein the powerful mechanical corrective impact of HTO itself dominates the 12-month postoperative functional recovery, masking the subtle clinical benefits of adjuvant biologic therapies.

However, second-look arthroscopy at 12 months unveiled a distinct biological narrative. Beyond mechanical correction, reduced intra-articular pressure has been shown to promote spontaneous, although partial, cartilage repair [28,29]. Our findings support this observation, as Group A (HTO alone) demonstrated measurable cartilage repair 12 months after surgery without adjuvant therapy. However, this intrinsic repair was limited, with a median Koshino staging score of 2.0 (IQR, 2.0–2.0) (Table 4). Most patients in Group A exhibited only with scattered fibrocartilage, indicating that although HTO creates a favorable environment for self-repair [25,28], biological stimulation alone may be insufficient for optimal structural restoration.

In contrast, Group B (LR-PRP) achieved a significantly superior cartilage repair appearance compared to both Group A and Group C (p<0.0001; Table 3 and Table 4). While Group C (LP-PRP) showed modest repair compared to the control group, its effectiveness appeared limited, even though Group C possessed less severe baseline defects than Group B. This suggests that the biological efficacy of the adjuvant therapy plays a more critical role in joint surface restoration than the initial lesion severity. Although HTO alone typically offers a joint survival rate of 10 to 15 years before a patient inevitably requires conversion to total knee arthroplasty, the significantly enhanced structural coverage and tissue quality achieved in the LR-PRP group may hold the potential to delay further joint degeneration, theoretically extending the long-term survival rate of the natural knee joint.

To enhance cartilage repair and address chronic inflammation, regenerative therapies such as PRP and MSCs have gained increasing attention. However, their clinical application remains challenged by issues related to standardization, dosing, and activation protocols [30]. The adoption of automated preparation systems, which complete processing in under 10 min, has helped reduce operator-dependent variability and improve reproducibility. In our clinical practice, the vast majority of patients received only a single postoperative intra-articular injection of PRP on the first postoperative day due to local economic considerations and insurance coverage. While multiple-injection protocols are frequently documented, our findings suggest that even a single-dose application of standardized LR-PRP can trigger a beneficial local healing response.

The superior cartilage repair appearance observed in the LR-PRP group may be attributed to the biological activity of leukocytes and their interaction with platelet-derived growth factors. PRP contains multiple bioactive molecules, including TGF-β, PDGF, and VEGF, which are essential for chondrocyte proliferation, extracellular matrix production, and angiogenesis [31,32]. Although the optimal leukocyte concentration remains debated [33,34], our findings indicate a beneficial effect of LR-PRP. In addition to delivering growth factors, leukocytes—particularly macrophages—may theoretically play a key role in modulating the intra-articular microenvironment. Macrophages can transition from a proinflammatory M1 phenotype to an anti-inflammatory and pro-regenerative M2 phenotype [35,36], a process essential for resolving inflammation and initiating tissue remodeling. Since no molecular, cellular, or immunological analyses were performed in the current study, these biological mechanisms remain strictly hypothetical and must be interpreted with extreme caution.

Several limitations should be considered. First, the sample size was relatively small and unevenly distributed among groups, which may reduce statistical power. Furthermore, this single-center retrospective design may introduce selection bias. Specifically, the strict exclusion of candidates who received other concurrent adjuvant therapies or who lacked mandatory 12-month second-look arthroscopy may have influenced group comparability and could potentially limit the generalizability (external validity) of our findings to broader clinical populations. Second, although second-look arthroscopy provided direct assessment of cartilage repair appearance at 12 months, this represents only midterm follow-up. Moreover, because the evaluation of cartilage repair was based purely on arthroscopic macro-appearance during hardware removal without histological biopsies, advanced biochemical assessments, or quantitative MRI cartilage mapping, we cannot verify true histological cartilage regeneration. Third, we must acknowledge a major confounding variable: LR-PRP and LP-PRP were processed using two entirely different commercial preparation systems. Consequently, the observed differences in structural repair cannot be solely attributed to the presence or absence of leukocytes; they may also be influenced by differences in absolute platelet dosage, processing methodologies, plasma composition, or residual red blood cell concentrations. Finally, the role of leukocytes in PRP remains highly controversial, with both proinflammatory and anti-inflammatory roles reported. Therefore, these findings should be interpreted cautiously, and further mechanistic, prospective, and standardized clinical trials are warranted to clarify the precise molecular pathways.

## 5. Conclusions

In conclusion, while HTO effectively and uniformly alleviates knee pain and restores physical function through mechanical realignment, the adjunctive use of LR-PRP is associated with a significantly superior arthroscopic appearance of cartilage repair compared to LP-PRP or HTO alone. A notable discrepancy was observed between subjective and structural outcomes; post-operative clinical pain and functional improvements (VAS, WOMAC, and OKS scores) were comparable across all groups, whereas the LR-PRP group demonstrated superior joint surface restoration with more complete defect coverage and tissue continuity.

However, given our retrospective design, small sample size, and the lack of histological validation, these findings must be interpreted cautiously as positive associations rather than direct causal effects or true histological regeneration.

Further well-designed prospective studies and randomized controlled trials with larger cohorts are needed to validate these findings and clarify the underlying biological mechanisms, ultimately supporting the development of standardized postoperative management protocols.

## Figures and Tables

**Table 1:** Basic information of the groups.

	Group A	Group B	Group C	Exact *p*-Value
Sex (F/M), *n*	6/4	12/8	7/3	0.852
Age (year), means ± SD (95% CI)	53.8 ± 12.46 (44.78 to 62.62)	60.4 ± 7.57 (56.85 to 63.95)	63.6 ± 8.59 (57.45 to 69.75)	0.061
Height (cm), means ± SD (95% CI)	164.88 ± 8.83 (158.56 to 171.20)	161.85 ± 10.34 (157.01 to 166.69)	158.6 ± 9.75 (151.63 to 165.57)	0.371
Weight (kg), means ± SD (95% CI)	71.83 ± 14.88 (61.19 to 82.47)	73.35 ± 14.4 (66.61 to 80.09)	63.54 ± 10.48 (56.04 to 71.04)	0.183
BMI (kg/m^2^), means ± SD (95% CI)	26.29 ± 4.28 (23.23 to 29.35)	27.86 ± 3.73 (26.12 to 29.61)	25.16 ± 2.52 (23.36 to 26.96)	0.152
Pre-HTO K-L grade, median (IQR)	3.5 (3.0–4.0)	3.5 (3.0–4.0)	3.5 (3.0–4.0)	1.000
Pre-HTO ICRS Grade, median (IQR)	4.0 (4.0–4.0)	4.0 (4.0–4.0)	3.0 (3.0–4.0)	0.0053

**Table 2:** Pre- and post-high tibial osteotomy operation evaluation for the knee.

	Group A	Group B	Group C	Exact *p*-Value	Effect Size (Partial *η*^2^)
Pre-HTO operation					
VAS score, mean ± SD (95% CI)	9.1 ± 0.74 (8.57 to 9.63)	9.35 ± 0.67 (9.04 to 9.66)	8.8 ± 0.63 (8.35 to 9.25)	0.122	--
WOMAC score, mean ± SD (95% CI)					
Pain	18.9 ± 1.66 (17.71 to 20.09)	19.25 ± 2.67 (18.00 to 20.50)	17.90 ± 1.66 (16.71 to 19.09)	0.3072	--
Stiffness	7 ± 1.25 (6.11 to 7.89)	6.4 ± 1.1 (5.89 to 6.91)	6.4 ± 1.51 (5.32 to 7.48)	0.426	--
Physical function	51.4 ± 3.31 (49.03 to 53.77)	53.1 ± 5.66 (50.45 to 55.75)	50.5 ± 5.95 (46.24 to 54.76)	0.412	--
OKS, mean ± SD (95% CI)	13.6 ± 2.76 (11.63 to 15.57)	11.35 ± 3.59 (9.67 to 13.03)	14.8 ± 3.49 (12.3 to 17.3)	0.029 *	--
Post-HTO operation					
VAS score, mean ± SD (95% CI)	0.9 ± 0.74 (0.37 to 1.43)	0.85 ± 1.14 (0.32 to 1.38)	0.6 ± 0.7 (0.1 to 1.1)	0.9541 ^	0.0029
WOMAC score, mean ± SD (95% CI)					
Pain	1 ± 0.82 (0.42 to 1.58)	1.15 ± 0.75 (0.80 to 1.50)	1.7 ± 1.77 (0.44 to 2.96)	0.5198 ^	0.0401
Stiffness	1.8 ± 0.79 (1.24 to 2.36)	1.55 ± 0.76 (1.19 to 1.91)	1.1 ± 0.99 (0.39 to 1.81)	0.1684 ^	0.1054
Physical function	3.9 ± 1.85 (2.57 to 5.23)	3.5 ± 1.93 (2.60 to 4.40)	2.8 ± 1.81 (1.50 to 4.10)	0.4966 ^	0.0428
OKS, mean ± SD (95% CI)	40.4 ± 2.95 (38.29 to 42.51)	41.85 ± 2.78 (40.55 to 43.15)	43.3 ± 2.63 (41.42 to 45.18)	0.1980 ^	0.0963

^ indicates that the post-HTO operation *p*-value was calculated using a multivariate covariate analysis (ANCOVA model), with age, gender, BMI, preoperative K-L, preoperative ICRS, and corresponding preoperative baseline scores as covariates for adjustment. * indicates a statistically significant difference (*p* < 0.05).

**Table 3:** Evaluation of ICRS grade in articular cartilage before and after High tibial osteotomy. Arthroscopic evaluation showed that up to 65% of patients in Group B (LR-PRP) regressed to a near-normal Grade 1.0 state, with a statistically significant difference between groups (*p* < 0.0001).

ICRS Repair Grade	Group A	Group B	Group C	Exact *p*-Value
Pre-HTO operation, *n* (%)				0.0053
Grade 3	1 (10%)	2 (10%)	6 (60%)	
Grade 4	9 (90%)	18 (90%)	4 (40%)	
Median (IQR)	4.0 (4.0–4.0)	4.0 (4.0–4.0)	3.0 (3.0–4.0)	
Post-HTO operation, *n* (%)				<0.0001
Grade 1	0 (0%)	13 (65%)	0 (0%)	
Grade 2	5 (50%)	7 (35%)	6 (60%)	
Grade 3	5 (50%)	0 (0%)	4 (40%)	
Median (IQR)	2.5 (2.0–3.0)	1.0 (1.0–2.0)	2.0 (2.0–3.0)	

**Table 4:** Evaluation of articular cartilage repair using the Koshino staging system. Koshino’s newborn coverage data shows that Group B had 55% complete Stage 4 coverage, while the other two groups had 0% (Exact *p* < 0.0001).

Koshino Grade, *n* (%)	Group A	Group B	Group C
1	0 (0%)	0 (0%)	0 (0%)
2	8 (80%)	0 (0%)	4 (40%)
3	2 (20%)	9 (45%)	6 (60%)
4	0 (0%)	11 (55%)	0 (0%)
Median (IQR)	2.0 (2.0–2.0)	4.0 (3.0–4.0)	3.0 (2.0–3.0)

## Data Availability

The raw data supporting the conclusions of this article will be made available by the authors on request.

## Supplementary Materials

The following supporting information can be downloaded at: https://www.mdpi.com/article/10.3390/biomedicines14081664/s1, Table S1: STROBE Statement checklist for observational cohort studies.

## Abbreviations

The following abbreviations are used in this manuscript:

ACL	Anterior cruciate ligament
ANCOVA	Analysis of covariance
EGF	Epidermal growth factor
HKA	Hip–knee–ankle
HTO	High tibial osteotomy
ICRS	International Cartilage Regeneration & Joint Preservation Society
IQR	Interquartile range
KL grades	Kellgren–Lawrence (K-L) grades
LP-PRP	Leukocyte-poor platelet-rich plasma
LR-PRP	Leukocyte-rich platelet-rich plasma
MSC	Mesenchymal stem cells
OA	Osteoarthritis
OKS	Oxford Knee Score
PCL	Posterior cruciate ligament
PDGF	Platelet-derived growth factor
PRP	Platelet-rich plasma
TKA	Total knee arthroplasty
VAS	Visual Analog Scale
VEGF	Vascular endothelial growth factor
WOMAC	Western Ontario and McMaster Universities Osteoarthritis Index
